# Coffee as a Disinfecting Agent

**Published:** 1848

**Authors:** 


ARTICLE XIII—*Coffee as a Disinfecting Agent.* By WEBER.

Coffee is one of the most powerful means not only of rendering animal and vegetable effluvia innocuous, but of actually destroying them. A room in which meat, in an advanced degree of decomposition, had been kept for some time, was instantly deprived of all smell on an open coffee roaster being carried through it, containing a pound of coffee newly roasted. In another room, exposed to the effluvium occasioned by the clearing out of a dung pit, so that sulphuretted hydrogen and ammonia in great quantity could be chemically detected, the stench was completely removed within half a minute on the employment of three ounces of fresh-roasted coffee; whilst the other parts of the house were permanently cleared of the same smell by being traversed with the coffee roaster, although the cleansing of the dung-pit lasted several hours longer. Even the smell of musk and castoreum, which cannot be overpowered by any other substance, is completely dispelled by the fumes of coffee; and the same applies to the odour of assafœtida. It was remarked, however, that, in general, animal effluvia are more readily affected by it than vegetable.

That here an actual neutralization, and not a mere envelopment of matter takes place, is shown from this—that the first fumes of the coffee are imperceptible, and continue so until a point of saturation, so to speak, is reached, whereupon the obnoxious smell disappears, and that of coffee predominates. The reverse happens with other aromatic vapours, and even with acetic acid and chlorine. Here both co-exist until the one completely preponderates. The simplest form to use it against contagious matter is in powder. The well-dried bean is to be pounded in a morter, and to be strewed over a moderately-heated iron plate, until the powder assumes a dark-brown tint. Caffeic acid, and the empyreumatic coffee-oil, act more readily in minute quantity. *Med. Gaz.* in Braith's Ret.

